# Supplementary material for: Development and validation of the Michigan Chronic Disease Simulation Model (MICROSIM)
Source: PLoS One. 2024 May 16;19(5):e0300005. doi: 10.1371/journal.pone.0300005 (PMC11098406; doi:10.1371/journal.pone.0300005)
Supplement: Attachment — Submitted filename: plosOne_responseToRevs_2.docx [file pone.0300005.s001.docx]

We thank the editors for their invitation to revise and resubmit our paper. We also thank the reviewer, Professor Krueger, for his detailed and thoughtful comments. We have responded to the comments below and, as suggested by the reviewer, we have substantially expanded and restructured the manuscript. We believe the manuscript has been substantially strengthened  through this process.

**Reviewer Comments**

Reviewer #1: I see that this is a paper outlining the construction of a modeling framework with the intent of allowing simulation-based approaches in exploring population-level impacts of different types of interventions. The current version of the code-base allows for interventions specifically around blood pressure and other risk factors. The intended benefits are to provide a tool that can supplement more traditional approaches, like RCTs, that provide strong evidence but have large, sometimes impossible, requirements given the possible health questions.

**Comment #1:** Let me start by saying that I strongly appreciate the focus on software design best practices. Pair programming, solid testing patterns, etc are vital for building the kind of complicated software projects that could be so valuable when trying to understand the many inter-related issues in population and public health. So thank you for voicing this.

***Response #1:*** *Thank you for this comment.*

**Comment #2:** A small first point is that the model name, MICROSIM, is almost the same as the common short-form for micro-simulation modeling. I would suggest changing the model name to avoid confusion.

***Response #2:*** *We’ve already presented results from this model under this name in abstract form and within our multi-institution research team, so we’re somewhat hesitate to change it for that reason. More generally, while we see the reviewer’s point about possible confusion we think that’s sufficiently unlikely to necessitate a name change. First, we think that the use of all capitals in the name is a common and accepted convention for acronyms that it should help most readers understand that the name is an acronym and not a literal description of the package, particularly as it is not uncommon for acronyms to refer to the content areas from which they arise. Second, the name intends to communicate the scope of questions that MICROSIM could help answer. While this initial iteration focuses on the specific motivating questions outlined in this manuscript, we designed MICROSIM to potentially answer similar chronic disease management questions across different populations, conditions, and treatments.*

**Comment #3:** A larger point, though, is that this is not what is typically understood to be an agent-based model. A key part to an ABM is some kind of interaction, either with other agents, with a shared environment, or both. And a key goal is generally to look for some kind of emergent property at the population level. As far as I can tell, this is certainly an individual-based model, but there is no inter-agent interaction, nor a shared environment. I don’t see either of these in this model.

Also, in ABMs, elements of the domain being modeled are generally labelled as endogenous, exogenous, or ignored. Endogenous elements are those whose mechanism is captured in some way in the model (e.g., an SEIR model of infection describes an infection mechanism). In MICROSIM, all dynamics are determined using cohort variables (e.g., age, sex, risk factors) and various statistical correlations derived from regression models. In other words, it sounds like each "person" is effectively a lone Markov process where the annual transition probabilities between different observable disease or risk states are determined using regression-based estimates. This could be described as an individual-based model, a micro-simulation model, or a Markov chain model, but it isn’t an agent-based model.

***Response #3:*** *Thank you for this helpful feedback. We have struggled to find the optimal words to describe the model. The primary “agent-like” features of the modeling approach are that: 1.) A relatively detailed phenotype defines each individual; 2.) Changes in the phenotype are tracked across the life course; and 3.) Complex interactions between individual phenotype elements may occur (e.g., trade-offs between mortality and dementia). But, as the reviewer correctly points out, MICROSIM lacks some common features of agent-based models, most notably direct interaction amongst agents.*

Our primary reason not to label MICROSIM as an “individual-based” model was because of the model’s focus on population-level effects and the specific inclusion of population-level recalibration for treatment effects. Individuals in populations not receiving blood pressure treatment are not entirely separate Markov processes. MICROSIM tabulates cardiovascular events at the population level at the end of each simulation wave. Then, MICROSIM nullifies or generates additional cardiovascular events to maintain observed population-level treatment effects. While it might be possible to implement treatment effects as part of an individual-level Markov process, we opted against that strategy because we felt that our approach allowed direct incorporation of the best available data into the model. Epidemiologic cohort studies give us the best evidence of untreated risk, and trials give us the best evidence of relative treatment effects. Our approach directly incorporates cohort-based risk models to estimate untreated risk and trial-based treatment effects at the population level.

Thus, we have opted to refer to our model more generically as a “population and individual-level micro-simulation model.” While lacking succinctness, we feel this description most accurately characterizes the model. We also think that this description helps elaborate both the rationale and strengths of MICROSIM more clearly.

**Comments #4:** A good resource that describes agent-based and other models is a report called “Best Practices for Systems Science Research” on the OBSSR website, available here: <https://obssr.od.nih.gov/sites/obssr/files/inline-files/best-practices-consensus-statement-FINAL-508.pdf>

***Response #4:*** *Thank you for sharing this reference. We have generally tried to align the language and structure of this paper with the guidance in that report.*

**Comment #5:** Now, as a Markov chain model, it is unclear to me how current methods of regression are unable to provide the same kinds of insight that are purported to be generated from this model. I appreciated the section called “Illustration of The Potential Utility of Agent-Based Model Framework” that seeks to highlight a specific example of value added by this model. But I am unable to judge the level of that contribution. Could one not already glean that population-level impacts are likely to be much lower than group-level variance by just using the regression-based hazards? What does MICROSIM add to this? I would find it helpful to see this compared with what could be done without the model to better highlight its unique contributions.

***Response #5:*** *Thank you for this helpful comment.*

*In retrospect, we did not do a great job articulating the point of that example. As the reviewer correctly points out, estimating between- and within-group variance can easily be done with regression approaches in a cohort study. Similarly, mean population-level effects can readily be estimated using more rigidly structured decision analytic techniques (e.g., using population-level mean probabilities to estimate state transitions instead of individual-level probabilities conditioned on covariates). MICROSIM’s design enables it to estimate population-level means based on real-world population parameters and between- and within-group variance at the individual level. In principle, this structure enables exploration of between- and within-group effects that may meaningfully differ from those estimated in cohort studies. For example, if there is marked uncertainty in the age-outcome association in older age groups and a cohort study undersamples those age groups relative to the target population, then a regression may underestimate the population-level uncertainty, and MICROSIM would be able to account for that type of difference.*

*We have reworked the text of the motivating example to articulate that the combination of estimating between- and within-group variation* ***and*** *mean population-level effects distinguishes MICROSIM from other modeling frameworks.*

**Comment #6:** More broadly, I don’t see how this model offers more than what’s already available with regression-based statistical approaches. I would benefit from other examples on how this model has been used, or can be used, to provide useful insights, especially if they are compared to current state-of-the-art approaches. And if it was already described in the text but I missed the relevant sections, I would benefit from an indication of which sections I missed and an expansion on that context. I was very intrigued by this sentence: “We can directly reuse the MICROSIM code in the trial assessment simulations to assess trial designs.” This is an area I think could be really approachable by simulation models. But I would need to see solid examples to motivate the use of this framework.

***Response #6:*** *We have added several clarifications.*

*First, we have added a section, “Overview of the Research Purpose and Specific Motivating Study Questions,” that articulates some of the motivating questions this model is designed to address and are not readily addressable by existing simulations.*

*Second, we have articulated that a key novel element of MICROSIM is that the simulation structure is conceptually well-aligned with the best available data sources for its purpose (Added to the first two paragraphs in the section “Overview of Simulation Evidence Evaluation Hierarchy and Structure”).*

*Third, we describe how researchers can apply MICROSIM to trials. In brief, an existing MICROSIM subpackage, “trials” has been developed that researchers can use to describe clinical trials and common trial parameters, execute trials using the MICROSIM codebase, and abstract trial result data for analysis. We are in the process of completing a separate manuscript describing this process and the results of specific trials, although that work is not yet complete.*

**Comment #7:** It also strikes me that there are already other well established packages for doing agent-based modeling. (I know I already argued that this isn’t ABM, but if something can do ABM, it can also do individual-based Markov chain models.) A presentation of a new tool kit that is built to occupy a role that is arguably already being filled by, say, Repast, NetLogo, or AnyLogic should do some work to compare it against the ABM tools already available, especially since those tools also allow the creation of proper ABM internal mechanism. What does this offer that current ABM frameworks don’t?

***Response #7:*** *We must admit that we are not experts in those specific packages but suspect that it would possible to answer some of the questions that MICROSIM is designed to address using those packages. The biggest difference between MICROSIM and those packages is its scope. Rather than being designed for a single questions (or set of narrowly related questions) as with most decision analyses or as a general purpose simulation structure (as the reviewer references) it is designed to be adaptable to using the use the best available evidence for a variety of chronic disease management questions.*

*In addition to that general difference, based on our best understanding of those packages, we believe that the primary virtue of MICROSIM is its design to use “inputs” readily available in existing clinical and epidemiologic data. (Elaborated in Paragraph 2 under Overview of Simulation Evidence Evaluation Hierarchy and Structure) Population-level recalibration to enable direct incorporation of trial-based treatment effects is not easily accomplished in these existing frameworks. It also appears that these frameworks focus on specifying agent-agent interaction and not on complex within-agent changes, which is the primary focus of MICROSIM (e.g., using different regression-based models to model risk factor change and outcomes over time).*

**Comment #8:** Perhaps a better organization of this work is for the main report to better motivate the model, with strong examples of how this offers unique value over the statistical methods required to build the model, and over current ABM modeling frameworks. The technical descriptions of which parts were estimated in what specific way, of the order of simulation steps, etc could be put into a supplementary technical report or users manual. (There are ways of describing ABMs, such as the PARTE or ODD frameworks, linked in that OBSSR report, but one could also provide more traditional software design approaches like class diagrams.)

I recognize that my requests would likely require quite substantial re-writes, but I think they would be necessary.

***Response #8:*** *We appreciate this helpful feedback and have restructured and rewritten the manuscript to better align with the “Best Practices for Systems Science Research” reference in comment one and to describe the “Simulation Details” sections using the PARTE framework.*

*We have retained the simulation details in the main manuscript for now. As this is a methods paper, it seemed reasonable to retain the details in the body of the manuscript, even though it has become rather lengthy. We would be happy to move the details to an appendix if the editorial team believed that was a more effective and efficient way to present this work.*

**Comment #9:** What are the computational details of this package? E.g., what is the memory footprint for running the examples in the paper?

***Response #9:*** *This varies quite substantially depending on the specific inputs and computational environment. But, the outcome validation simulation takes used about 10 GM of RAM while running for 12 hours to run on a single core of a machine with Intel Xeon 8268 Cascade Lakes 2.9 GHz CPU. We are working on strategies to optimize parallelization.*

**Comment #10:** I strongly appreciate the goal of improve transparency. How does one do that for non-coders? You had to go out to industry to find someone with software design experience. I’m not sure I see how this solves the black-box science barrier for the majority of the professional audience of this work.

***Response #10:*** *We agree that this would not address the black-box science problem for non-coders tackling a novel problem. Our idea is that, by designing MICROSIM as an extensible framework, it may be possible for less sophisticated coders to get the virtues of our development process (including professional software expertise) by either directly operating within the existing framework or, alternatively, extending the framework to address questions related to chronic disease management. We added text to the “Simulation Design Principles” section: “We hope that by designing a simulation following these principles, simulation reliability and accuracy will be enhanced. We further hope that designing an extensible framework potentially adaptable to various chronic disease management problems will enable researchers to build upon this framework to maximize the reliability and validity of subsequent analyses.”*

**Comment: #11:** It was stated in several places that a key metric of success is how “accurate” this model is, but how accurate can it be if it’s parameterized using survey data? Is this even a useful goal for such models?

**Response #11:**

*First, given the many uses of the word “accuracy” we have reworded some of our uses — particularly whether the simulation produces results consistent with those observed from similar high-quality data that was not incorporated in the models ‘design. So, for example, incidence of stroke and MI is thought to be optimally measured with disease-specific population-based cohort studies. MICROSIM fairly reasonably reproduced those data using completely separate survey and cohort-based inputs, increasing our confidence that it is likely to accurately inform the key trade offs that it is designed to assess. We generally retained the word “accuracy” when referring to the overall simulation goals at a high level.*

*We understand the reviewer’s general point but believe that accuracy against survey data is still important. While survey data is imprecise, it provides a standard, grounding results to the best available estimates for assessing population-level health . While survey data has some measurement error, it is also the gold standard for assessing population-level health. Both NHANES and the cohort studies apply gold-standard measurement or the key elements including in MICROSIM, standardized protocols and population-based sampling.*

**Comment #12:** In several places, the text describes how to trigger an event with certain probability (e.g., “by determining whether a [0, 1) random uniform number was below that partition threshold”). I think much of this text could be cut out and replaced with just “[some property] was assigned with a certain probability”. I think it would make the text cleaner.

***Response #12:*** *Thank you for this suggestion. We have implemented it throughout and agree it considerably simplified the text.*

**Comment #13:** What is an “event-partitioning model”? The link is only the Github page with the python code.

**Response #13:** *It is not a term of art, but rather a descriptive term that we coined for a model to predict the probability that an ASCVD event was a stroke or an MI. We have removed reference to this term in the revised manuscript.*

**Comment #14:** Shouldn’t table 2 have uncertainty bounds of some type to allow better comparison?

**Response #14:** *We certainly can include various confidence intervals, but intentionally omitted them from this table as the primary determinant of the confidence interval width in the simulation is the arbitrarily large sample size we used to initialize the validation simulation.* *Moreover, given the substantial variation in gold-standard estimates we concluded that bounds of simulation estimates wouldn’t likely alter our conclusions regarding how well the results correspond with external standards.*

**Comment #15:** Table 3 should have the empirical values included for easier comparison.

***Response #15:*** *We have made this change.*

**Comment #16:** There are a few spelling or grammar errors that can be easily fixed (e.g., the word “xcluded”)

***Response #16:*** *We have fixed that error and re-read the manuscript to address other spelling or grammar issues.*
