# Supplementary material for: Development and validation of the Michigan Chronic Disease Simulation Model (MICROSIM)
Source: PLoS One. 2024 May 16;19(5):e0300005. doi: 10.1371/journal.pone.0300005 (PMC11098406; doi:10.1371/journal.pone.0300005)
Supplement: Attachment — Submitted filename: plosOne_secondResponseToRevs.docx [file pone.0300005.s002.docx]

Dear Dr. Sipilä and Dr. Krueger-

Thank you again for considering our manuscript. Responses to Dr. Krueger’s critiques are outlined below. We genuinely believe the manuscript has been strengthened through review and the productive feedback.

Thanks,

Jim Burke

**Reviewer #1: The changes to the manuscript were very welcomed. I think the paper makes a strong contribution. I only have a suggestion for 1 more addition: What are the assumptions that are core to MICROSIM?**

**There are many necessary assumptions in the use case in this paper, but many of them are related to those of the sub-models (i.e., those statistical models within the "Model Repository"). Since this framework is intended to showcase a general approach, different sub-models can be used as evidence changes. There's no need in my eyes to changing this in the current manuscript.

However, I think there are 2 types of assumptions that seem to be related to the structure of MICROSIM. And, in my mind, these deserve special mention, even if briefly. One is that causal assumptions are needed to link some intervention(s) and some outcome(s). In this case I believe it's assumed that BP medication directly influences CVD and CVD events and nothing else. There are no side effects on any other relevant properties of the person. And it's assumed that BP medication impacts GCP and dementia indirectly through CVD and CVD events. There's no direct causal link between BP medication and GCP/dementia. Of course with greater evidence, these could be improved, but a set of "causal assumptions" that go beyond the data seems to be always needed.

And there are assumptions based on how the population is "aged" over time. With PARTE framework language, one of the "actions" is that some non-clinical properties (e.g., BMI) are updated at each model update point. How is this done? (This is the "rule" part. There's no problem to keeping these in the same paragraph.) Also, this means that someone taking BP medication will also see changes to other of their properties depending on how this update was done. Does it, for example, create the assumption that the history of BP doesn't directly impact future outcomes since it's only using the current state? Or that a reduction in BP through medication has the same future trajectory as having a naturally lower BP? Again these assumptions no doubt could be changed, but there always needs to be some kind of assumption like this.

I would find a brief paragraph about each would be useful, even in the discussion section, so that future users can be clear about the kinds of assumptions necessitated by this approach.**

**Response:**

Thank you for this helpful feedback. We have added a section at the end of the “Simulation Details” subsections that summarizes the core assumptions of MICROSIM: “*Simulation Details: Summary of Core Assumptions”.*

The text of this section is reproduced in italics for convenience:

*Simulation Details: Summary of Core Assumptions*

*The central baseline assumption of MICROSIM is that it reflects the US population at any given time point in the 21^st^ century. Thus, the population, baseline levels of risk and unmeasured factors giving rise to both change in risk factors and outcomes over time reflect the average state of affairs in the US during this epoch. These assumptions are embedded in both the population-level data used to initialize the simulation and the data driving the underlying risk and outcome models selected for MICROSIM. A key implication of this assumption is that “usual care” reflects care that was delivered in the United States at a give time. While central, the MICROSIM framework enables this assumption to be relaxed or modified by either initializing with a different set of population parameters and/or changing the risk factor/outcome models.*

*Several key assumptions regarding BP treatment are built into MICROSIM. BP treatment is assumed to have narrow and specific effects, specifically: 1. Lowering SBP and DBP by a standard average effect (5.5/3.1 mm Hg) and have a resultant small effect in reducing the risk of ASCVD events via this level of BP reduction 2. Further reducing ASCVD event risk, such that the overall relative risk reduction of BP medications reflects trial meta-analyses (relative risk, RR, of 0.79/BP medication for stroke and RR of 0.87/BP medication for MI) 3. Improving GCP and reducing dementia risk mediated only through the effect of BP lowering. As such, the effect of a BP lowering medication on GCP is mediated entirely through the effect of lowering BP by 5/3 mm Hg and the effect on dementia is mediated entirely through the GCP effect.*

*Treatment effects operate across the life course. When a BP treatment is applied, it reduces a simulated person’s BP at a given point in time. When the person is “aged” forward, the treated BP is used to predict the subsequent BP via risk factor model updating. Thus, the effect of BP treatment over time may have larger or smaller effects over time, based on the details of the risk factor updating models. This assumption can be modified within the existing MICROSIM treatment framework by specifying alternate BP treatment effects (e.g. reducing BP every wave on treatment by 5/3 mm Hg, without updating subsequent risk factors), but the default is to apply a BP treatment at one wave and then subsequent update BP via changes in risk factor models over time.*

In addition, we have the following to clarify the question of how treatment effects how individuals are “aged” over time in the subsection, “*Simulation Details: Rules — Treatment Effects”:*

“Blood pressure treatments can be applied in MICROSIM in one of two ways — they can either be applied at a single point in time or they can be reapplied during each simulation wave. In both cases, SBP and DBP in subsequent waves will reflect the lower BP in the prior wave. In this way, it is possible to model different assumptions about the life course effects of BP medications. Default behavior is to apply a treatment at a given point (e.g. add one medication to lower BP by 5.5/3.1 mmHg in a given wave) and then to have the lower BP level in that wave predict subsequent BP levels via risk factor updating.”
